# Metabolomic Analysis of Complex Chinese Remedies: Examples of Induced Nephrotoxicity in the Mouse from a Series of Remedies Containing Aristolochic Acid

**DOI:** 10.1155/2013/263757

**Published:** 2013-03-31

**Authors:** Dong-Ming Tsai, Jaw-Jou Kang, Shoei-Sheng Lee, San-Yuan Wang, I-Lin Tsai, Guan-Yuan Chen, Hsiao-Wei Liao, Li Wei-Chu, Ching-Hua Kuo, Y. Jane Tseng

**Affiliations:** ^1^Graduate Institute of Biomedical Electronics and Bioinformatics, National Taiwan University, No. 1, Sec. 4, Roosevelt Road, Taipei 106, Taiwan; ^2^The Metabolomics Core Laboratory, Center of Genomic Medicine, National Taiwan University, No. 1, Sec. 4, Roosevelt Road, Taipei 106, Taiwan; ^3^Institute of Toxicology, College of Medicine, National Taiwan University, No. 1, Sec. 4, Roosevelt Road, Taipei 106, Taiwan; ^4^Department of Pharmacy, College of Medicine, National Taiwan University, No. 1, Sec. 4, Roosevelt Road, Taipei 106, Taiwan; ^5^Department of Computer Science and Information Engineering, National Taiwan University, No. 1, Sec. 4, Roosevelt Road, Taipei 106, Taiwan; ^6^Sheng Chang Pharmaceutical Co., Ltd., Jung-Li, Taiwan

## Abstract

Aristolochic acid nephropathy is caused by aristolochic acid (AA) and AA-containing herbs. In traditional Chinese medicine, a principle called “Jun-Chen-Zou-Shi” may be utilized to construct a remedial herbal formula that attempts to mitigate the toxicity of the main ingredient. This study used *Bu-Fei-A-Jiao-Tang* (BFAJT) to test if the compound remedy based on a principle of “Jun-Chen-Zou-Shi” can decrease the toxicity of AA-containing herbs. We compared the three toxicities of AA standard, *Madouling* (an *Aristolochia* herb), and a herbal formula BFAJT. AA standard was given for BALB/c mice at a dose of 5 mg/kg bw/day or 7.5 mg/kg bw/day for 10 days. *Madouling* and BFAJT were given at an equivalence of AA 0.5 mg/kg bw/day for 21 days. Nephrotoxicity was evaluated by metabolomics and histopathology. The urinary metabolomics profiles were characterized by ^1^H NMR spectroscopy. The spectral data was analyzed with partial least squares discriminant analysis, and the significant differential metabolites between groups were identified. The result showed different degrees of acute renal tubular injuries, and metabolomics analysis found that the kidney injuries were focused in proximal renal tubules. Both metabolomics and pathological studies revealed that AA standard, *Madouling*, and BFAJT were all nephrotoxicants. The compositions of the compound remedy did not diminish the nephrotoxicity caused by AA.

## 1. Introduction

Aristolochic acids (AAs) are potent nephrotoxic agents [1, 2] that are found primarily in the plant genera *Aristolochia* and *Asarum *[3, 4]. These herbs have been used as a component of herbal remedies in traditional Chinese medicine (TCM). Herbal remedies containing *Aristolochia* and *Asarum* have been used to relieve symptoms such as cough, arthritic pain, and gastrointestinal problems [5, 6]. However, chronic kidney injury may occur in humans after a prolonged intake of these Aristolochiaceous herbs. Inadvertent replacement of *Stephania tetrandra* by *Arsitolochia fangchi* has caused rapidly progressive interstitial renal fibrosis (also named Chinese herbs nephropathy) in young women on a slimming regimen [7, 8]. AAs were determined to be the major components that caused the toxicity [9]. The kidney injury induced by AA-containing herbs characterized by tubulointerstitial injury and paucity of filtration of inflammatory cells in the kidney is named aristolochic acid nephropathy (AAN) [10, 11]. Until prohibited, these herbs were widely used worldwide and victims of AAN had been reported in many countries [2, 8, 12].

TCMs are generally used as compound remedies which are composed of several herbals. Chinese herbal classics indicate that the components in herbal remedies can be divided into the 4 principles: “Jun-Chen-Zou-Shi” which represents “the emperor, the minister, the assistant and the courier” [13, 14]. The emperor herbs (Jun) are the main components to relief symptoms. The minister herbs (Chen) act as an adjunct to facilitate the emperor herbs in relief of symptoms. The assistant herbs (Zou) help to enhance the efficacy provided by Jun and Chen and to counteract toxic and side effects caused by these herbs. The courier herbs (Shi) act as an emollient for the herbal remedy [15]. AA-containing herbs are generally used as compound remedies. Many studies have been performed on the nephrotoxicity of pure aristolochic acid, but there is very limited nephrotoxicity information regarding the commonly used medicinal herbs of Aristolochiaceae or the compound remedies containing the Aristolochiaceae herb. Before AA was outlawed, several herbal formulas containing AA had been used in TCM. Longdan Xieganwan is a TCM formula, which contains *Caulis Aristolochiae manshuriensis* among its 10 ingredients. The remedy was used as a “liver enhancer” and its toxicity was supposed to be lessened via the combination of other components according to the Jun-Chen-Zou-Shi theory. However, Londan Xieganwan had been reported to be toxic in humans and rats [16–18]. These observations made the concept of using compound remedies to reduce AAN a debatable issue. Another herbal formula *Bu-Fei-A-Jiao-Tang* (BFAJT) which is a decoction containing *Fructus Aristolochia contorta* (*Madouling*) is used for some lung-related symptoms [19]. No study discusses the toxicity of this herbal formula until now.

Metabolomics is a newly developed technology to study phenotype changes of the cellular responses to pathophysiological stimuli or genetic modification through a holistic metabolite analysis [20, 21]. The metabolite profile comprises hundreds to thousands of endogenous organic metabolites. Through analytical platforms such as proton nuclear magnetic resonance (^1^H-NMR) or hyphenated liquid chromatography with mass spectrometry (LC-MS), metabolite profiles can be obtained [22, 23]. With the advance of computers and chemometric techniques, the complex data resulting from these platforms can be mined for useful information [24].

Several studies have applied a metabolomics approach to study AA toxicity. Zhang et al. reported that AA given rats showed significant renal toxicity with a metabolite pattern similar to other proximal renal tubular toxicants through ^1^H NMR spectroscopic metabolomic study [25]. Liang et al. used ^1^H NMR to study renal toxicity of *Aristolochia fangchi* in rats, and the AA equivalent dose they used was 3.7 mg/kg/day for 4 weeks. Renal toxicity was detected at 2 weeks in their study [26]. Chen et al. used LC-MS to investigate AA and *Aristolochia manshuriensis* nephrotoxicity in rats, and the equivalent AA dose of *A. manshuriensis* they used was 96 mg/kg/day for 4 consecutive days. They indicated that a metabolomics approach is promising in providing rapid screening of nephrotoxicity [27].

To test if the compound remedy based on a principle of “Jun-Chen-Zou-Shi” can decrease the toxicity of AA containing herbs, we used BFAJT as our test remedy to study its nephrotoxicity by metabolomics. The advantages of using ^1^H-NMR experiment in metabolomics include simple sample preparation and high system robustness [28]. It detects the resonance signal of different proton groups and can provide the structural information of metabolites. ^1^H-NMR was applied to obtain the urinary metabolic profiles of mice treated with herbs. This study anticipates providing scientific evidence of nephrotoxicity of BFAJT.

## 2. Materials and Methods

### 2.1. Animal Handling and Sampling

Animal care and handling protocols were in compliance with national animal treatment guidelines and approved by the Animal Committee of National Taiwan University. All animal studies were performed in the animal center of National Taiwan University Medical College Animal Center. A total of 24 male BALB/*c *mice aged 6–8 wk (18–20 g) were obtained from the Laboratory Animal Center, Medical College of National Taiwan University, Taipei, Taiwan. Regular rodent laboratory chow (Purina Mills, Inc., St. Louis, MO) and water were allowed freely. Animals were lodged in individual metabolic cage and acclimated in temperature 25°C and humidity 60% with regular day/dark light cycle, starting from one week before each experiment to reduce the stress of adjusting to new environment for animals. Same conditions were used throughout the experiments.

### 2.2. Chemicals and Herbal Materials

Authentic pure reference aristolochic acid, *Madouling* (*Fructus Aristolochia contorta*), and a compound remedy Bu-Fei-A-Jiao-Tang (BFAJT) were used in this study. Aristolochic acid was purchased from Acros Organics (NJ, USA). The content is AA-I 96% (90.9%) and AA-II 4% (5.7%). AA-I is the major constituent of AAs in our test standard and it is also the major aristolochic acid component in the tested herb. Therefore, an AA-I equivalent dose was used to control the AA administration dose for mice fed with AA standard, Madouling, and BFAJT. *Madouling* powder was purchased from Sheng Chang Pharmaceutical Co., Ltd. (Chung-Li, Taiwan). The dried decoction powder was filtered and extracted from boiled herb. The dosing sample was a mixture of the decoction and corn oil. The content of AA-I is 24.17 mg/gm and of AA-II is 2.04 mg/g for the dosing sample. BFAJT powder was purchased from Sheng Chang Pharmaceutical Co. The dried decoction powder was processed using the same procedure as that of *Madouling*. The content of AA-I is 3.749 mg/g and of AA-II is 0.169 mg/g for the dosing sample. The BFAJT powder is composed of donkey hide gelatin 45 g, *Madouling* 15 g, apricot seed 6 g, great burdock fruit 7.5 g, rice 30 g, and honey fried licorice root 7.5 g.

### 2.3. HPLC Conditions

The equipment consisted of a pair of ShimadzuLC-10 AT pumps (Kyoto, Japan), a Rheodyne 7725i5-mL manual injector (Cotati, CA, USA), and a Shimadzu SPD-M10A diode array detector. Separations were carried out on a Luna C column, 250∗4.6 mm, 5 *μ*m (Phenomenex, Torrance, CA, USA). The mobile phase was composed of 0.7% acetic acid and acetonitrile, 57 : 43 (v/v).

### 2.4. Experiment Design

The experiments were divided into two parts. Experiment 1 investigated the toxicity of AA reference standard. Nine mice were randomly divided into 3 groups. They were control group (*n* = 3) treated with vehicle of corn oil, the middle dosed group (*n* = 3) treated with AA 5 mg/kg bw per day, and the high dosed AA group (*n* = 3) treated with AA 7.5 mg/kg bw per day. The three groups were tagged as AA0, AA5, and AA7.5. AA was dissolved in corn oil with a concentration of 1 and 1.5 mg/mL. The vehicle and AA were given to mouse via oral gavage once daily. Urine samples were collected on days 1, 3, 8, and 10 after dosing. The collected urine was centrifuged at 3000 rpm for 15 min immediately, and the clear suspension was stored at −80°C after adding sodium azide to reach a final concentration of 10 mM of sodium azide. All mice were euthanized after experiment for renal histopathological analysis at 10 days after dosing. Urine samples were sent for NMR analysis. Body weights were measured on selected days.

Experiment  2 investigated toxicity of AA containing herbals in low AA dosage. The equivalence dose of AA for both *Madouling* group and BFAJT group is 0.5 mg/kg bw per day. Nine mice were randomly divided into three groups; they were control group (*n* = 3), treated with vehicle, *Madouling* dosed group (*n* = 3), treated with *Madouling* powder 400 mg/kg bw per day, and BFAJT dosed group (*n* = 3), treated with BFAJT 4 g/kg bw per day. The 3 groups were tagged as C0, M0.5, and BF0.5. The equivalent amount of AA for *Madouling* 400 mg and BFAJT 4 g is 0.5 mg. All substances were dissolved in corn oil and given through oral gavage once daily. Collection and handling of urine was similar as described in the first part experiment. Urine samples collected at days 1, 3, 10, and 13 were sent for ^1^H NMR spectroscopy. All mice were euthanized at day 20 after dosing, and a histopathological study of the kidneys was performed. A summary of these two experiments is described in Table 1.

### 2.5. Renal Histopathology

The section of formalin-fixed paraffin-embedded kidney tissue was stained with hematoxylin/eosin. The stained kidney sections were analyzed under a light microscope. The degree of renal lesions was graded from one to five depending on the severity: 1 = minimal (<1%); 2: slight (1%–25%); 3 = moderate (26%–50%); 4 = moderate/severe (51%–75%); 5 = severe/high (76%–100%) [29]. It was according to the renal histopathological findings of anatomical site of lesion (cortex to medulla), location of renal tubular lesion (proximal to distal, focal to locally extensive), morphology of renal tubular lesion (dilatation with or without hyaline cast to necrosis), and patterns of inflammation (acute to subacute).

### 2.6. NMR Spectroscopic Analysis of Urine

^1^H NMR spectroscopy was performed from collected urine samples. A test sample of 825 *μ*L for each mouse was prepared using 500 *μ*L of the urine sample, 250 *μ*L of 0.2 M Na_2_HPO_4_ (pH 7.4), and 75 *μ*L of sodium 3-trimethylsilyl-1-(2, 2, 3, 3-*d*_4_)propionate (TSP) in D_2_O (final concentration 0.1 mg/mL). D_2_O provided an NMR lock signal for the NMR spectrometer. Conventional ^1^H NMR spectra of the urine samples were obtained from a Bruker Avance 600 spectrometer (Bruker Biospin, Germany) operated at 600.04 MHz at 25°C. One-dimensional ^1^H NMR spectra were acquired using a standard NOESYPR1D pulse sequence (recycle delay-90°-*t*_1_-90°-*t*_*m*_-90°-acquisition; XWIN-NMR3.5) with a recycle delay time of 2 s, and a mixing time of 150 ms. The 90° pulse length was adjusted to ~12.5 *μ*s at −1 dB and *t*_1_ was set to 3 *μ*s, which provided an acquisition time of 2.72 s. The FIDs were multiplied by an exponential weighting function corresponding to a line broadening of 0.3 Hz, and the data were zero-filled to 64 k data points. All spectra were corrected for phase and baseline distortions and referenced to the internal reference standard TSP (*δ* ^1^H = 0.0). Each ^1^H NMR free induction decay (FID) data was transformed to 1D spectrum in ACD/Labs v10.0 1D NMR manager (Advanced Chemistry Development, Inc., Canada). The spectral data was exported to a 16 k data points text file recording chemical shifts and their respective signal intensities. Baseline correction and binning were performed using an in-house script under the *R* statistical environment (version 2.11.1) [30]. The spectral intensities were binned in 0.04 ppm from 0 ppm to 10 ppm and scaled. Intensity data of water (4.5-5.5 ppm) and urea (5.5–6.0 ppm) were set to zero. To normalize metabolite concentration among these spectra, a probabilistic quotient normalization algorithm was performed [31].

### 2.7. Multivariate Analysis

Partial least squares discriminant analysis (PLS-DA) is a common approach to multivariate metabolomics data analysis. PLS analysis maximizes the product of variance matrix of measured variables (e.g., NMR metabolomic profile data) and correlation of measured data with properties of interest (e.g., toxicity), while DA predicts class membership of a dataset X with a y vector including only 0 and 1 (1 indicates that one sample belongs to a given class). For more than 2 classes, the PLS2 algorithm was applied [32]. PLS-DA was performed using the pls package (version 2.10) [33] in R. Validation of PLS-DA classification models was performed by cross model validation using the method of Westerhuis et al. [34]. In addition, a permutation test is applied with 2,000 random assignments of classes. The test set sample classification errors were evaluated to qualify the classification results. Scoring plots with two components were drawn for spectral classification. Loadings plots were drawn to search for significant chemical shift variables. To evaluate the fitness of the model, values of explained variation, *R* ^2^ > 0.7, and predicted variation, *Q* ^2^ > 0.4 is considered as a good model [35].

Urine metabolites were assigned by referencing peak pattern and chemical shift from the NMR library of Human Metabolic Database (HMDB) [36], Chenomx NMR Suite Professional software package version 7.0 (Chenomx Inc., AB, Canada) and previous reports on rodent urine ^1^H NMR in the literature [37, 38].

Paired univariate binned NMR data between groups was analyzed with a nonparametric Wilcoxon rank-sum test. Bins with *P* value less than 0.05 were considered as significant metabolites.

## 3. Results and Discussion

The experimental design is shown in Table 1. Experiment 1 was designed as the positive control, and the two dosing groups of 0, 5, and 7.5 mg/kg bw/day of AA standard in mice. HPLC was used to quantify the content of AAs in Madouling and BFAJT. Experiment  2 was designed to evaluate the nephrotoxicity of AAs containing herbs. The contents of AA-I and AA-II in Madouling are 1.051 and 0.089 *μ*g mg^−1^, respectively. The contents of AA-I and AA-II in BFAJT are 0.113 and 0.012 *μ*g mg^−1^, respectively. AA-I is the major constituent of AAs in our test standard, and it is also the major aristolochic acid component in the tested herb. Therefore, an AA-I equivalent dose was used to control the AA administration dose for mice fed with AA standard, Madouling and BFAJT. Considering the daily maximum feeding amount for mice, the AA-I equivalent doses for Madouling, and BFAJT groups were 0.5 mg/kg bw/day in Experiment  2.

### 3.1. Physiological Changes and Pathology

**Figure 1:**
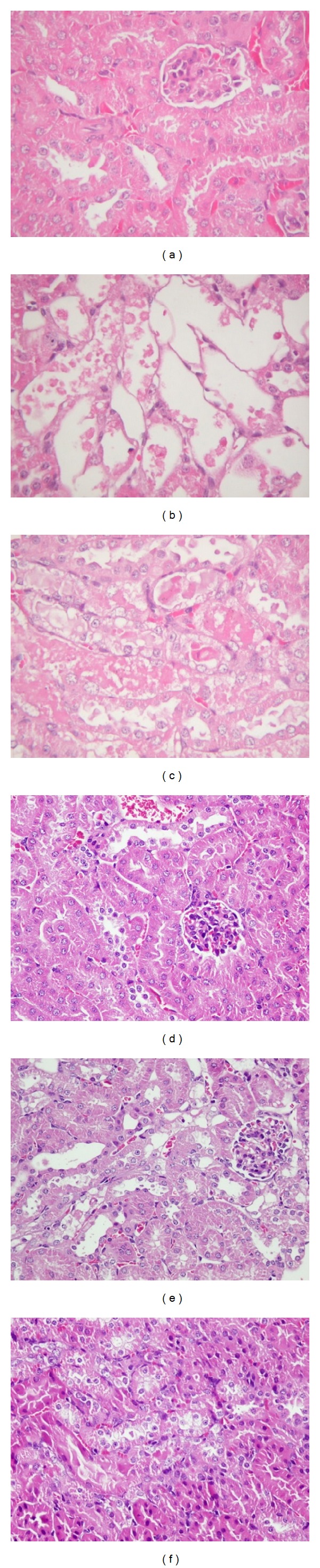
Representative histology (HE stain, magnification 400x) of kidney tissue from the different treatment groups. At 10 days in Experiment 1 ((a)–(c)), no significant alterations of renal tubules and glomeruli in control group (a). After treatment with medium dose of AA (5 mg/kg/day), kidneys revealed moderate to severe acute proximal tubular necrosis (b). After treatment with high dose of AA (7.5 mg/kg/day), kidneys showed moderately to severe acute proximal tubular necrosis (c). At 20 days in Experiment  2 ((d)–(f)), no significant alterations of renal tubules and glomeruli in control group (d). After treatment with Madouling, the kidney showed focal, slight acute proximal tubular degeneration with cellular swelling (e). The change in BFAJT showed acute proximal tubular hydropic degeneration which is similar to mice treated with Madouling (f) (both with AA dosage equivalent to 0.5 mg/kg/day).

The body weight of mice treated with different doses of AA standard showed no significant changes from day 0 to day 9. Renal pathology revealed that the control group (treated with vehicle only) showed normal morphology on day 10. Mice treated with AA standard and herbals showed kidney injuries of different degrees. In Experiment 1, both AA standard treated groups (AA5 and AA7.5) showed a grade 3-4 severe shrinking of the proximal tubular cytoplasm and atrophy plus luminal dilation of the distal tubular system in large parts. Despite this acute renal tubulointerstitial injury, the glomerular morphology was relatively preserved in the AA dosed groups, and inflammatory cell infiltration was not prominent (Figures 1(a), 1(b), and 1(c)). This pathological change is similar to other AAN studies both on humans and mice [2, 39].

In Experiment  2, after 20 days of vehicle treatment, normal renal pathohistology was observed for the control group. For mice treated with Madouling, the proximal renal tubules showed slight acute tubular degeneration and cellular swelling focally. In the BFAJT treated group, kidneys of mice showed similar mild proximal renal tubular injury as in the Madouling group. In all groups, no significant glomerular changes were observed (Figures 1(d), 1(e), and 1(f)). In summary, under treatment of high dose of AA standard (AA 5.0 mg/kg bw/day or higher), the kidney showed severe acute renal tubulointerstitial injuries. For mice treated with Madouling and BFAJT (AAI equivalent dose 0.5 mg/kg bw/day), the renal tubular lesions showed mild change at day 20 for both groups. In this study, the accumulative dosage of AA standard to induce acute renal histopathological changes was 50–75 mg/kg bw, which was equivalent to LD50 reported by Mengs given by a single oral dose [1]. Renal tubular atrophy and interstitial fibrosis were also observed by other studies with intraperitoneal injection of AA [39, 40]. Shibutani et al. tested the mouse by orally administered 2.5 mg/kg/day of AA-I and found severe renal tubular injuries with little interstitial inflammation at 10 days [41]. Compared to Experiment 1, the acute AA nephropathy was minor in Experiment  2 due to lower AA administration dose. The administration dose of AA in Experiment  2 was restricted by maximum feeding amount for mice, since the contents of AAI in Madouling and BFAJT were only 1.051 and 0.113 *μ*g mg^−1^, respectively. Even though the administration dose was low, renal tubular lesions were still observed in kidneys.

### 3.2. Metabolic Changes in Urine Samples by ^1^H-NMR

**Figure 2:**
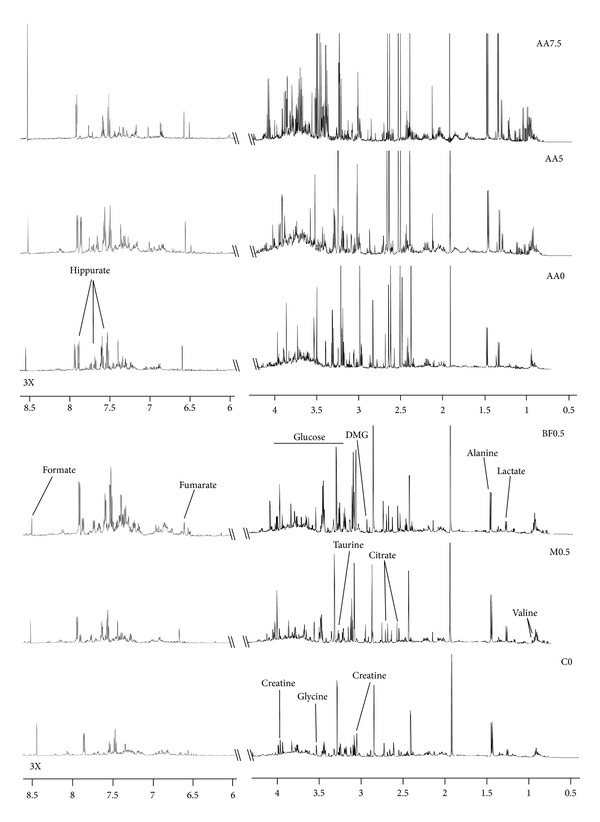
Representative ^1^H NMR spectra of mouse urine after treatment for 13 days. Signals are assigned to their respective metabolites. The aromatic region (*δ* 6.0–8.5) was magnified three times in signal intensity as compared to the aliphatic region (*δ* 0.5–4.5).

Mice urine was collected in each group on different days of the experiment (Table 1). The urine samples were subjected to ^1^H NMR analysis to investigate the metabolic changes in urine caused by AA treatment. Representative 600 MHz ^1^H NMR spectra from control and dosed groups are shown in Figure 2. The NMR spectra of mouse urine specimens showed different metabolic pattern after treatment for 10 days and 13 days in Experiment 1 and in Experiment  2, respectively. Multiparametric statistical analysis was applied to analyze ^1^H-NMR spectra and to investigate the differential metabolites between control and AA treated groups.

### 3.3. Multiparametric Statistical Analysis of ^1^H-NMR Data

**Figure 3:**
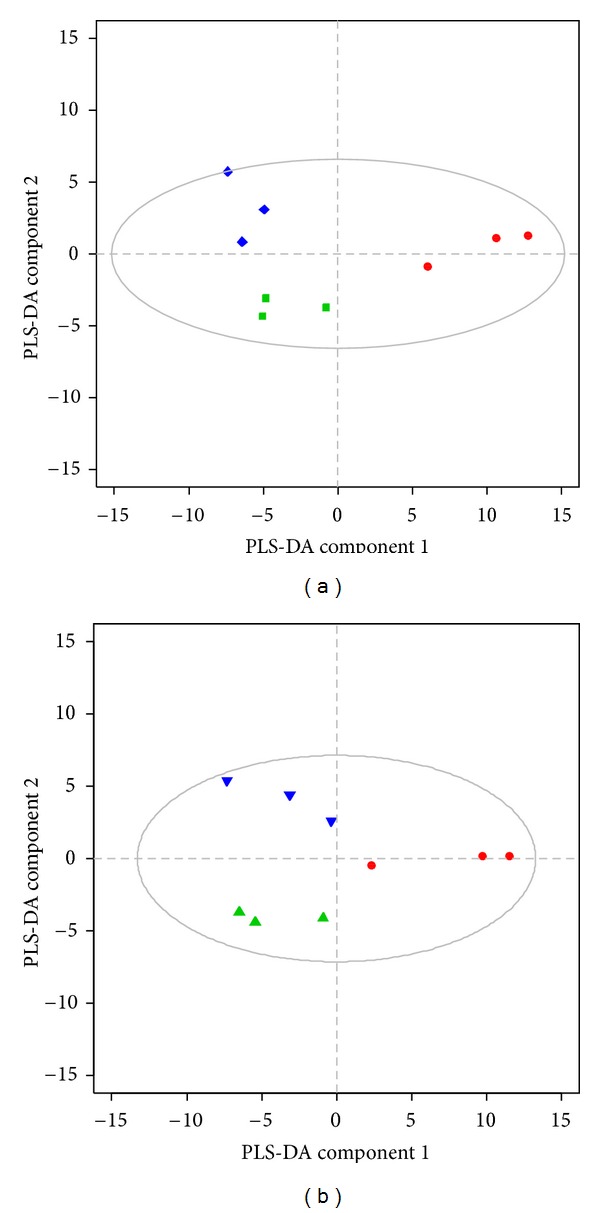
Partial least squares discriminant analysis (PLS-DA) scores plot showing clustering of different dosing groups using urinary ^1^H-nuclear magnetic resonance (NMR) dataset at day 10 in Experiment 1 (a) and at day 13 in Experiment  2 (b). Data symbols: AA0 *⚫* (red), AA5 ▪ (green), and AA7.5 *◆* (blue) of Experiment 1, C0 *⚫* (red), M0.5 ▲ (green), and BF0.5 *▼* (blue) of Experiment  2. The ellipse represents Hotelling's T2 with 95% confidence.

A PLS-DA model was constructed to characterize the relationship among mouse groups.  Figure 3 shows the first two components of the PLS-DA scores plots for both experiments. In Experiment 1, PLS-DA scores plots showed a good separation between the AA dosed groups (AA5, AA7.5) and control group (AA0) along the component 1 axis on day 10. We further used *R* ^2^ and *Q* ^2^ parameters to discriminate and predict the metabolic pattern difference between every two groups. In the scores plot, the *R* ^2^ value represents the percent variance we extracted from the spectral data and the *Q* ^2^ value represents the group predictability. Here, we showed the group discrimination of AA5 versus AA0, also for AA7.5 versus AA0 (the *R* ^2^, and *Q* ^2^ values between AA0 and AA5 are 0.88 and 0.50; the *R* ^2^ and *Q* ^2^ values between AA0 and AA7.5 are 0.94 and 0.71). But the discrimination between AA7.5 and AA5 is poor (the *R* ^2^, and *Q* ^2^ values between AA5 and AA7.5 are 0.85 and −1.03) as the *Q* ^2^  value is negative. The findings of multivariate analysis with PLS-DA show a compatible group difference as found in renal pathology (Figure 3(a)). In Experiment  2, a scores plot of PLS-DA showed clustering of the three groups (C0, M0.5, and BF0.5) at day 13 (Figure 3(b)). The *R* ^2^ and *Q* ^2^ values between C0 and M0.5 are 0.80 and 0.23, while the *R* ^2^ and *Q* ^2^ values between C0 and BF5 are 0.77 and 0.12, showing underlying metabolic perturbation between dosed groups and control group. However, these separations between the two dosed groups M0.5 and BF0.5 are weak (a negative *Q* ^2^ value of −0.65 and *R* ^2^ value of 0.97). Compared to the pathological changes in Figure 1, we can discriminate between control group and dosed groups at even an earlier stage using PLS-DA prior to the pathological proof.

### 3.4. Metabolite Change in Influenced Pathway

**Figure 4:**
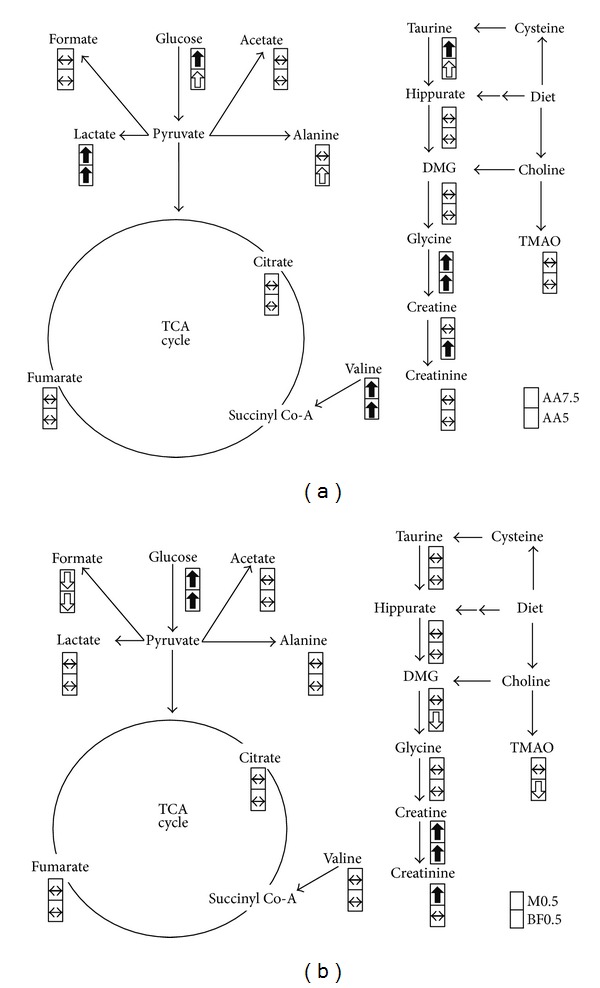
Perturbed metabolic pathways in response to AA substance exposure. (a) Energy metabolism, several amino acids, and creatinine are influenced. The metabolite concentrations are significantly increased for lactate, glucose, taurine, glycine, valine, and creatine for AA5 and AA7.5 on days 8–10 in Experiment 1. (b) Changes for M0.5 and BF0.5 on days 10–13 in Experiment  2. DMG dimethylglycine, TMAO trimethylamine-N-oxide. Symbols in the cell represent relative concentration change of assigned metabolite between groups. They are significant increase (the black arrow up word)/decrease (the black arrow down word), *P* < 0.05; nonsignificant increase (the white arrow up word)/decrease (the white arrow down word), fold change > 2; no significant difference (↔).

Loadings plots were drawn to search for significant chemical shift variables. After identifying differential chemical signals, metabolites were assigned according to those chemical signals by Chenomx NMR Suite. Resonances with different intensity between the dosed and control groups were assigned to creatine, glycine, creatinine, TMAO (trimethylamine-N-oxide), valine, hippurate, DMG (dimethylamine), citrate, lactate, alanine, glucose, fumarate, and formate (Figure 4). These metabolites and their relevant metabolic pathway were investigated for the underlying acute kidney injury from AA intoxication.

Detection of increased glucose and lactate in urine may suggest injuries in proximal renal tubules by nephrotoxicants. In the AA standard treated group, we observed an increase in glucose and lactate concentration in urine. In the *Madouling* and BFAJT treated groups, we found an increase in glucose concentration in urine, but the concentration of lactate did not show significant change. A number of nephrotoxicants have been studied by metabolomics [42–45]. In gentamicin-induced nephrotoxicity in rats, there was increased in the concentration of glucose and lactate in urine [42, 43]. As the lesion of gentamicin is mainly on the proximal renal tubule, characterized by a marked epithelial necrosis, the increase of glucose concentration in urine denotes perturbed proximal renal tubular reabsorption. Besides, the increased lactate concentration in urine indicates the loss of epithelial mitochondrial function. In a study of region specific nephrotoxins in rats, the increased concentration of glucose and lactate in urine was found in toxicants that injure the proximal renal tubule, such as hexachlorobutadiene, HgCl_2_, and sodium chromate, but this phenomenon was not found in nephrotoxicants that damage the region of renal papilla, such as propylene and 2-bromothanamine hydrobromide [44]. Since glucose and lactate concentration increased in AA treated groups in this study, it may suggest that the lesions caused by AA were on the proximal renal tubules. The metabolomic observation is correlated to the observation in the histopathological examination. In the M0.5 and BF0.5 groups, only glucose concentration was elevated in urine which may suggest a minor degree of proximal tubular lesion as we detected in the histopathological examination. In other metabolomic studies of AA nephrotoxicity, increase in glucose and lactate concentration in urine accompanying renal proximal tubular lesions has been detected in rats [25, 26]. Another study indicated that the AA acute kidney injury caused diffuse degeneration of the proximal tubular epithelium [39].

Creatine was increased in the AA dosed groups. Increased creatine concentration in urine has been reported in subclinical renal papillary injury by 2-bromoethanamine hydrobromide [45]. The increase in creatine level in urine might be due to a variety of factors including creatine reabsorption, cell leakage, changes in both muscle mass, and bowel microflora metabolism [46]. The change in creatine concentration in this study may be related to renal papillary dysfunction with subclinical morphological change in the renal papillary region.

In herbal treated groups, both M0.5 and BF0.5 groups showed evidence of kidney injury from the changed concentration of glucose and creatine/creatinine. The similar trends with the AA standard group with lesser prominent changes of several metabolites (lactate, glycine and valine) may have resulted from the minor kidney injury.

In conclusion, AA standard, *Madouling,* and BFAJT were all nephrotoxicants as indicated by both metabolomics and pathological studies. The compositions of the compound remedy did not diminish the nephrotoxicity caused by AA. ^1^H-NMR was demonstrated as a convenient instrument to detect kidney injury, and it can be applied to evaluate the complicated metabolic response caused by herbal formulas. The control group and AA challenged groups can be classified by PLS-DA scoring plots of NMR spectra. The prediction strength from PLS-DA is stronger for the AA standard group as this group was administered higher amounts of AA. NMR metabolomics shows potential for early detection of AAN when coupled with multivariate pattern recognition analysis.

## Figures and Tables

**Table 1:** Experiment design.

Group^a^	Substance	Eq. dose to AA (mg/kg bw/day)	Urine sampling date	
Experiment 1			
AA0	Vehicle	0	Day 1, 3, 8, 10^b^	
AA5	AA	5.0	Day 1, 3, 8, 10^b^	
AA7.5	AA	7.5	Day 1, 3, 8, 10^b^	
Experiment 2		
C0	Vehicle	0	Day 1, 3, 10, 13^c^	
M0.5	*Madouling *	0.5	Day 1, 3, 10, 13^c^	
BF0.5	BFAJT	0.5	Day 1, 3, 10, 13^c^	

^a^Three mice/group. ^b^Mice of AA groups were euthanized on day 10 for renal histopathology.

^c^Mice of all groups were euthanized on day 20.

AA: aristolochic acid; BFAJT: *Bu-Fei-A-Jiao-Tang*.
